# Antiviral mechanism of glycyrrhizic acid against BVDV: suppression of viral replication and immune regulation

**DOI:** 10.3389/fmicb.2026.1924475

**Published:** 2026-10-29

**Authors:** Yunnan Fang, Youli Yu, Zhengqing Yu, Jiandong Wang, Zhengwei Zhao, Yanan Guo, Yuxi Zhao, Dongdong Cai, Yuqiu Yang, Pan Zhou

**Affiliations:** 1 Animal Science and Technology, Ningxia University, Yinchuan, Ningxia, China; 2 Institute of Animal Science, Ningxia Academy of Agricultural and Forestry Science, Yinchuan, China; 3 Animal Disease Control Center of Yanchi County, Yanchi, China

**Keywords:** anti-infection research, bovine viral diarrhea virus, glycyrrhizic acid, gut microbiota, mouse model

## Abstract

**Introduction:**

Bovine viral diarrhea virus (BVDV), a globally prevalent pathogen in cattle, causes severe economic losses through acute diarrhea, immunosuppression, and reproductive failure, with mortality rates reaching 20%–30% in persistently infected herds. This study investigated glycyrrhizic acid (GA), a bioactive triterpenoid glycoside derived from licorice root with known anti-inflammatory and broad-spectrum antiviral properties, as a potential inhibitor of BVDV replication.

**Methods:**

Using *in vitro* (MDBK cells) and *in vivo* (BALB/c mice) models, we evaluated the antiviral and immunomodulatory effects of GA. Cells were treated with 10 μM GA, and mice received 20 mg/kg/day GA. Viral replication, cytokine expression, hepatic histopathology, and gut microbiota were assessed.

**Results:**

GA treatment significantly reduced BVDV replication, decreasing 5’UTR and NS5B mRNA expression by >80% (*P* < 0.01) and viral titers by 2.3-log in liver tissue. GA concurrently modulated host immune responses, suppressing pro-inflammatory cytokines (TNF-α by 68%, IL-6 by 54%) while elevating IL-10 (2.1-fold), and substantially improved hepatic histopathology scores (*P* < 0.05 versus infected controls). ELISA revealed GA-mediated downregulation of IFN-β during infection. Analysis of gut microbiota at 7 days post-infection revealed that both GA and ribavirin (RBV) treatments modulated microbial community structure, with RBV showing profiles more closely resembling uninfected controls.

**Discussion:**

These findings establish GA’s dual antiviral mechanism—direct suppression of viral RNA synthesis and immunomodulation—positioning this natural compound as a promising therapeutic candidate against BVDV infection in livestock.

## 1 Introduction

Bovine viral diarrhea virus (BVDV), a member of the *Pestivirus* genus within the Flaviviridae family, is one of the most economically significant pathogens affecting cattle industries worldwide (Feng et al., 2025; Liu et al., 2019). The virus causes substantial losses through acute infections characterized by diarrhea, fever, and immunosuppression, as well as through persistent infections that lead to reproductive failure and congenital defects in calves. Global seroprevalence studies indicate infection rates of 60%–90% in cattle herds, with annual economic losses exceeding $3 billion due to reduced productivity, premature culling, and trade restrictions (Pinior et al., 2017; Richter et al., 2017). Current control strategies primarily rely on vaccination and the identification/elimination of persistently infected animals, as no specific antiviral therapies are commercially available (Kim et al., 2023; Makoschey et al., 2007; Zhang et al., 2022). While modified-live and inactivated vaccines exist, their efficacy is limited by viral genetic diversity and the inability to prevent fetal infections (Engle et al., 2024; Van Campen et al., 2024). These critical gaps underscore the urgent need for novel, safe antiviral agents against BVDV.

Natural products have emerged as promising sources of antiviral compounds due to their structural diversity and low toxicity profiles (Chunarkar-Patil et al., 2024). Glycyrrhizic acid (GA), the primary bioactive triterpenoid saponin derived from *Glycyrrhiza glabra* (licorice root) (Isbrucker and Burdock, 2006), has emerged as a promising candidate due to its established safety profile and broad-spectrum antiviral activity (Ojha et al., 2016; Sarwar et al., 2022; Zulfugarova et al., 2023). GA has been used for centuries in traditional medicine and is approved in several countries for clinical applications, with demonstrated safety in both humans and animals (Duan et al., 2014). *In vitro* studies consistently show GA exhibits low cytotoxicity, with concentrations up to 100 μM maintaining >90% cell viability in various cell lines (Maestrini et al., 2021). Animal studies further confirm its safety, with mice tolerating doses up to 50 mg/kg without adverse effects. Importantly, GA’s safety advantages over conventional antivirals like ribavirin - which causes hemolytic anemia and teratogenicity - make it particularly attractive for veterinary applications (Connor et al., 1993; Lertora et al., 1991; Mehta et al., 2020; Zarogiannis et al., 2012).

Glycyrrhizic acid has demonstrated potent antiviral effects against multiple viruses including SARS-CoV-2, hepatitis C virus (HCV), and respiratory syncytial virus (RSV) through diverse mechanisms (Francis et al., 2002; Lacaille-Dubois and Wagner, 1996; Singh et al., 2017). These include direct virucidal activity by inhibiting viral entry or replication, as well as immunomodulatory effects through suppression of NF-κB-mediated inflammation or enhancement of interferon responses. Notably, GA was shown to block SARS-CoV-2 replication by activating the cGAS-STING pathway, while its derivative disrupted HCV assembly by binding to the NS5B polymerase (Abaeva et al., 2023). Despite these advances, the potential of GA against BVDV remains unexplored, representing a significant gap in veterinary antiviral research.

Based on GA’s established safety profile and demonstrated broad-spectrum antiviral activity, we postulated that it might represent a novel therapeutic strategy against BVDV infection. Specifically, we hypothesized that GA would exert its anti-BVDV effects through a dual mechanism: (1) direct inhibition of viral replication by interfering with key viral components such as the 5’UTR genomic region or NS5B polymerase, and (2) modulation of host antiviral immunity through regulation of interferon and cytokine responses. To test this hypothesis, we employed a comprehensive experimental approach combining *in vitro* cell culture models with *in vivo* murine infection systems. This study represents the first systematic investigation of GA’s antiviral potential against BVDV, with particular emphasis on elucidating its molecular mechanisms of action. Our findings not only contribute to the fundamental understanding of GA’s antiviral properties but also provide crucial preclinical data supporting its potential application in BVDV control programs, offering a much-needed alternative to current limited therapeutic options.

## 2 Materials and methods

### 2.1 Reagents and cell culture

The Madin-Darby bovine kidney (MDBK) cell line (ATCC^®^ CCL-22™) was obtained from the preserved cell culture collection of the Animal Science Research Institute, Ningxia Academy of Agricultural and Forestry Sciences (Yinchuan, China). The cells were maintained in Dulbecco’s Modified Eagle Medium (DMEM; Beyotime Biotechnology, Shanghai, China) supplemented with 10% heat-inactivated fetal bovine serum (FBS; Beyotime Biotechnology, Shanghai, China), 100 U/mL penicillin, and 100 μg/mL streptomycin at 37 °C in a 5% CO_2_ humidified atmosphere. Phosphate-buffered saline (PBS; Beyotime Biotechnology, Shanghai, China), tissue fixation solution (Beyotime Biotechnology, Shanghai, China), and cell digestion solution (Beyotime Biotechnology, Shanghai, China) were used for cell culture maintenance and processing.

The cytopathic bovine viral diarrhea virus (BVDV) NADL strain was obtained from the preserved viral collection of the Animal Science Research Institute, Ningxia Academy of Agricultural and Forestry Sciences (Yinchuan, China). This strain was originally isolated from clinical cases and has been maintained through serial passages in MDBK cells to preserve its cytopathic characteristics. Prior to experimental use, the virus was propagated in MDBK cells for three passages to ensure consistent infectivity, followed by determination of viral titer using TCID_50_ assay on MDBK cell monolayers. Aliquots of the virus stock were stored at −80 °C until use. Glycyrrhizic acid (GA; purity > 99%; Aladdin Biochemical Technology, Shanghai, China) and ribavirin (RBV; purity > 99%; Aladdin Biochemical Technology, Shanghai, China) were dissolved in dimethyl sulfoxide (DMSO; Sigma-Aldrich, St. Louis, MO, USA) to prepare stock solutions, with the final DMSO concentration maintained below 0.1% in all experiments to ensure minimal solvent effects on cell viability.

### 2.2 Cytotoxicity assay (CCK-8)

Madin-Darby bovine kidney cells in logarithmic growth phase were seeded in 96-well plates at a density of 5 × 10^4^ cells/well (100 μL/well) and incubated overnight at 37 °C with 5% CO_2_. The cytotoxicity assay included three experimental groups: (1) blank control (medium only), (2) negative control (cells + medium), and (3) treatment groups exposed to either glycyrrhizic acid (GA) or ribavirin (RBV) at nine different concentrations (0.1, 1, 5, 8, 10, 15, 20, 25, and 30 μM). Each concentration was tested in eight replicate wells to ensure statistical reliability. After 24 h of drug exposure, cells were washed three times with PBS to remove residual compounds, followed by addition of 10 μL CCK-8 reagent (Dojindo, Kumamoto, Japan) per well. The plates were gently mixed and then incubated for 4 h in the dark at 37 °C to allow color development. Absorbance was measured at 450 nm using an ALLSHENG AMR-100 microplate reader (Hangzhou Allsheng Instruments Co., Ltd., Hangzhou, China). Cell viability was calculated using the formula: (OD_*treatment*_ − OD_*blank*_)/(OD_*negative control*_ − OD_*blank*_) × 100%, with concentrations maintaining >90% cell viability considered non-toxic and selected for subsequent antiviral experiments.

### 2.3 *In vitro* antiviral assays

The antiviral activity of GA against BVDV was evaluated using four experimental groups: (1) negative control (uninfected MDBK cells), (2) BVDV-infected control (MOI = 0.1), (3) BVDV-infected cells co-treated with 10 μM GA, and (4) BVDV-infected cells treated with 15 μM ribavirin (RBV) as a positive control. Cells and virus were harvested at 6, 12, 24, and 48 h post-infection (hpi) for time-course analysis. Viral RNA (targeting 5’UTR and NS5B regions) and host cytokine mRNA expression (IFN-β, TNF-α, IL-6) were quantified by qRT-PCR using SYBR Green chemistry (HiScript II One Step qRT-PCR SYBR Green Kit, Vazyme Biotech, Nanjing, China), with primer sequences listed in Table 1. The relative expression levels were normalized to the endogenous control β-actin and calculated using the 2^−ΔΔCt^ method. All experiments were performed in triplicate to ensure reproducibility.

**TABLE 1:** Primer sequence.

Primer name	Primer sequence 5′-3′
Bovine β-actin-F	TCGGTTGGATCGAGCATTCC
Bovine β-actin-R	ACAAAAGCGATCACCTCCCC
BVDV 5′UTR-F	CCCTCTGCGAAGGCCGAAAAG
BVDV 5′UTR-R	CGAACTCACCACTGTTGCTACCC
BVDV NS5B-F	CCATCATCCCACACCACCTAC
BVDV NS5B-R	TTCCTGCCCACTACATCCTTG
Mouse β-actin-F	GCAGGAGTACGATGAGTCCG
Mouse β-actin-R	ACGCAGCTCAGTAACAGTCC
Mouse TGF-β-F	CATTGCTGGTCCAGTCTGCTTC
Mouse TGF-β-R	TGGTGAATGACAGTGCGGTTATG
Mouse IL-17-F	ATGCTGTTGCTGCTGCTGAG
Mouse IL-17-R	GAGGTTGACCTTCACATTCTGGAG
Mouse IFN-γ-F	ATGGCTGTTTCTGGCTGTTACTG
Mouse IFN-γ-R	TTCTTCCACATCTATGCCACTTGAG
Mouse IL-10-F	TGCCAAGCCTTATCGGAAATGATC
Mouse IL-10-R	TTCTCACCCAGGGAATTCAAATGC
Mouse TNF-a-F	TGTCTACTCCCAGGTTCTCTTCAAG
Mouse TNF-a-R	GACGGCAGAGAGGAGGTTGAC
Mouse IL-6-F	GAGAGGAGACTTCACAGAGGATACC
Mouse IL-6-R	TCATTTCCACGATTTCCCAGAGAAC
Mouse IL-1b-F	TCCCAAGCAATACCCAAAGAAGAAG
Mouse IL-1b-R	GTGAGGTGCTGATGTACCAGTTG

### 2.4 *In vivo* mouse model

Forty female BALB/c mice (6–8 weeks old) were randomly divided into four experimental groups (*n* = 10 per group): (1) BVDV-infected group, (2) GA-treated group, (3) negative control group, and (4) RBV-positive control group. For downstream analyses, 6 mice per group were randomly selected for histopathological scoring, liver cytokine qRT-PCR, and serum cytokine/viral load measurements, while 5 mice per group were randomly selected for cecal microbiota analysis. Because liver, serum, and cecal samples could be collected from the same animal, the two subsets partially overlapped, and the total number of animals per group remained 10. All mice were maintained under specific pathogen-free (SPF) conditions throughout the study. For infection, mice in the BVDV-infected, GA-treated, and RBV-positive control groups received an intraperitoneal injection of BVDV NADL strain (1 × 10^8^ PFU/mouse in 500 μL PBS), while the negative control group received an equal volume of sterile PBS. At 24 h post-infection (hpi), treatment was initiated with GA (5 or 20 mg/kg/day in physiological saline) or RBV (20 mg/kg/day in physiological saline) administered via oral gavage once daily for 7 consecutive days. Mice were euthanized by anesthesia at 24 h after the final treatment (8 days post-infection). Liver and spleen tissues were collected for viral load analysis, and serum samples were obtained for cytokine profiling. For histopathological examination, liver tissues from 6 randomly selected mice per group were fixed in 4% paraformaldehyde for 24 h prior to hematoxylin and eosin (HE) staining.

Before tissue collection at the end of animal experiments, all BALB/c mice were anesthetized via intraperitoneal injection of pentobarbital sodium. The pentobarbital sodium with purity ≥99% was dissolved in sterile 0.9% normal saline to prepare a 50 mg/mL working solution, and the standardized anesthetic dosage was set as 50 mg/kg body weight for each mouse. After injection, mice were placed in quiet SPF cages for continuous observation; dissection and tissue sampling were only performed after complete loss of pedal withdrawal reflex and corneal reflex to ensure adequate anesthesia depth. After tissue collection under deep anesthesia, all mice received secondary intraperitoneal injection of pentobarbital sodium at an overdose dose of 150 mg/kg body weight for humane euthanasia in accordance with the AVMA Guidelines for the Euthanasia of Animals and ARRIVE 2.0 principles. Each mouse was observed for no less than 5 min after lethal injection, and permanent cessation of heartbeat, respiration and corneal reflex was verified to confirm complete death. All mouse carcasses were collected uniformly and treated by the institutional professional biosafety waste treatment agency complying with laboratory animal biosafety regulations. All animal procedures in this study were approved by the Animal Ethics Committee of Ningxia University (Approval No. NJAULLSC2024024) and carried out in strict compliance with the 3R principles of animal welfare.

### 2.5 Histopathological and immunological analyses

For histopathological evaluation, liver tissues were fixed in 4% paraformaldehyde and processed for histological analysis. Sections (4 μm thick) were baked at 60 °C for 1 h, deparaffinized in xylene I and II (10 min each), and rehydrated through a graded ethanol series (100%, 95%, 85%, and 70%). Tissue sections were stained with Harris hematoxylin (containing mercuric oxide) for 5 min, rinsed with running water, differentiated in 1% acid alcohol for 30 sec, and blued in running water for 15 min. Counterstaining was performed with 0.5% eosin Y for 2 min, followed by rapid dehydration and clearing in xylene. Mounted sections were examined under light microscopy (Nikon Eclipse Ci-L, Japan) to assess inflammatory cell infiltration (lymphocytes, neutrophils) and necrotic changes. Two independent pathologists, blinded to the treatment groups, performed histopathological scoring.

Interferon-beta (IFN-β) levels in cell culture supernatants and mouse serum were measured using a commercial bovine IFN-β ELISA kit (ELK Biotechnology, Wuhan, China). Briefly, 100 μL of samples or standards were added to antibody-coated wells and incubated for 90 min at 37 °C. After washing, biotinylated detection antibody was added (60 min, 37 °C), followed by HRP-streptavidin conjugate (30 min, 37 °C). The reaction was developed with TMB substrate for 15 min in the dark and stopped with 2M H_2_SO_4_. Absorbance was measured at 450 nm using an ALLSHENG AMR-100 microplate reader. Sample concentrations were interpolated from the standard curve (range: 15.6–1000 pg/mL), with inter- and intra-assay coefficients of variation <10%.

### 2.6 16 S rRNA gene sequencing and gut microbiota analysis

Cecal contents were aseptically collected from mice in each group [negative control, BVDV-infected control, GA treatment group (20 mg/kg), and RBV positive control (20 mg/kg)] at 7 days post-infection (dpi). Samples were immediately snap-frozen in liquid nitrogen and stored at −80°C until analysis (*n* = 5 mice per group for microbiota analysis).

Total microbial genomic DNA was extracted using the E.Z.N.A.^®^ Soil DNA Kit (Omega Bio-tek, USA) following the manufacturer’s protocol. DNA purity and integrity were verified by 1% agarose gel electrophoresis and NanoDrop 2000 (Thermo Fisher Scientific, USA). The V4-V5 hypervariable region of the bacterial 16S rRNA gene was amplified with universal primers: 515F (5′-GTGCCAGCMGCCGCGGTAA-3′) and 907R (5′-CCGTCAATTCMTTTRAGTTT-3′). PCR reactions were performed in a 25 μL volume containing 12.5 μL 2 × Taq Plus Master Mix, 1 μL each primer (10 μM), 2 μL template DNA, and 8.5 μL nuclease-free water. Cycling conditions: 95°C for 5 min; 30 cycles of 95°C for 30 s, 55°C for 30 s, 72°C for 30 s; final extension at 72 °C for 10 min.

PCR amplicons were purified using the AxyPrep DNA Gel Extraction Kit (Axygen Biosciences, USA) and quantified with the Quant-iT PicoGreen dsDNA Assay Kit (Invitrogen, USA). Paired-end sequencing was performed on the Illumina MiSeq platform (Illumina, USA) at Majorbio Bio-Pharm Technology Co., Ltd., (Shanghai, China). Raw reads were filtered and chimeras removed using QIIME2 (v2022.11). Operational Taxonomic Units (OTUs) were clustered at 97% similarity. Alpha diversity (Chao1, Shannon, Simpson indices) and Beta diversity (Bray-Curtis distance-based PCoA/NMDS) were analyzed using R (v4.2.0). Species composition was visualized at phylum and genus levels. Linear discriminant analysis Effect Size (LEfSe) was used to identify differentially abundant taxa (LDA score > 2, *P* < 0.05).

Functional prediction of gut microbiota metabolic potential was performed using Tax4Fun2 (v1.1.6) to link taxonomic data to functional pathways. Briefly, the filtered OTU table was aligned to the SILVA SSU rRNA database (v138) for taxonomic annotation, then mapped to the Kyoto Encyclopedia of Genes and Genomes (KEGG) database (v97.0) to predict functional gene abundance. Key metabolic pathways were prioritized for analysis, including those involved in short-chain fatty acid (SCFA) synthesis (e.g., Butanoate metabolism, Propanoate metabolism) and inflammation regulation (e.g., NF-κB signaling pathway, TNF signaling pathway). Relative abundances of KEGG pathways (Level 2 and Level 3) were normalized to total functional gene counts, and statistical differences between groups were determined using one-way ANOVA with Tukey’s *post-hoc* test (*P* < 0.05 considered significant).

### 2.7 Statistical analysis

All quantitative data are presented as mean ± standard error of the mean (SEM). Statistical comparisons were performed using one-way ANOVA with Tukey’s *post-hoc* test in GraphPad Prism 9.0 (GraphPad Software, San Diego, CA, USA). A *P*-value < 0.05 was considered statistically significant.

### 2.8 Ethics statement

All procedures using SPF BALB/c mice were approved by the International Society for the Evaluation and Accreditation of Laboratory Animal Management. The Animal Experimentation Ethics Committee of Nanjing Agricultural University, Nanjing, China (NJAULLSC2024024), approved all the animal experiments in this research.

## 3 Results

### 3.1 GA exhibits low cytotoxicity in MDBK cells

The cytotoxicity of GA and RBV on MDBK cells was evaluated using the CCK-8 assay to determine non-toxic concentrations for subsequent antiviral experiments. MDBK cells in the logarithmic growth phase were exposed to 9 gradient concentrations (0.1, 1, 5, 8, 10, 15, 20, 25, and 30 μM) of GA or RBV for 24 h, with 8 replicate wells per concentration. Cell viability was calculated as (OD_450_ of treatment group - OD_450_ of blank control)/(OD_450_ of negative control - OD_450_ of blank control) × 100%.

As shown in Figure 1A, GA at concentrations ≤10 μM had no significant cytotoxicity on MDBK cells, maintaining a cell viability of >95% (*P* > 0.05 vs. the untreated negative control). When the concentration exceeded 15 μM, GA began to exhibit mild cytotoxicity, with cell viability decreasing to ∼85% at 20 μM and further to <70% at 30 μM. For RBV (Figure 1B), a concentration of 15 μM was confirmed to be non-toxic, preserving >90% cell viability (*P* > 0.05 vs. negative control), while concentrations ≥20 μM led to a significant decline in cell viability (*P* < 0.05). Based on these results, 10 μM GA and 15 μM RBV were selected as the working concentrations for subsequent *in vitro* antiviral assays.

**FIGURE 1:**
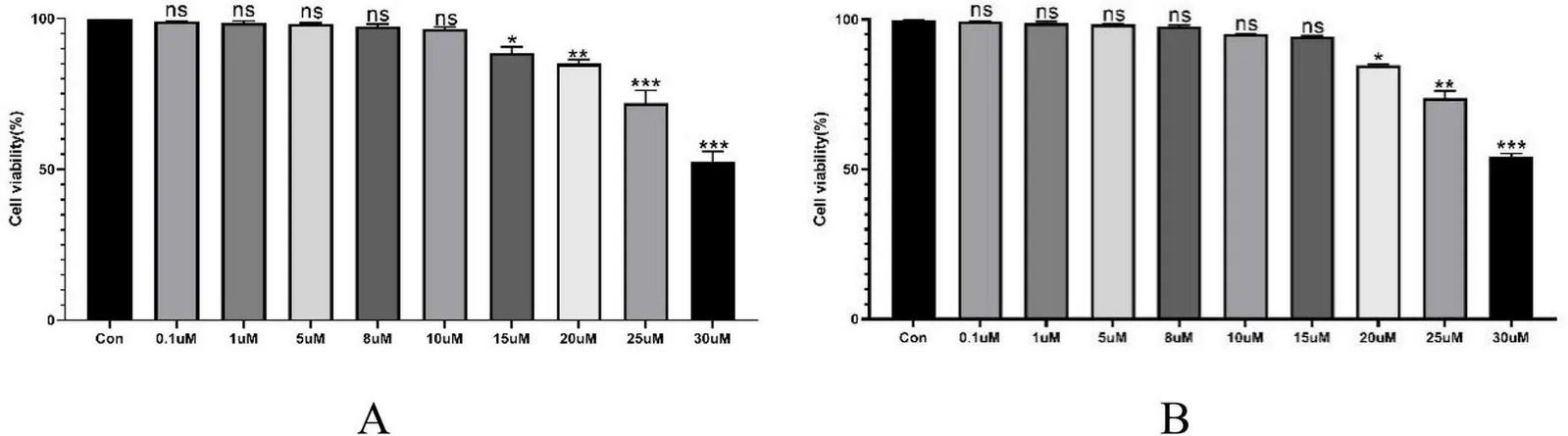
Cytotoxicity of glycyrrhizic acid (GA) and ribavirin (RBV) on MDBK cells. **(A)** Cytotoxicity of glycyrrhizic acid (GA) on MDBK cells. Logarithmic growth phase MDBK cells were treated with GA at nine gradient concentrations (0.1, 1, 5, 8, 10, 15, 20, 25, and 30 μM) for 24 h, with 8 replicate wells set for each concentration. Cell viability was detected using the CCK-8 assay, and calculated as [(OD_450_ of the treatment group - OD_450_ of the blank control) / (OD_450_ of the negative control - OD_450_ of the blank control)] × 100%. **(B)** Cytotoxicity of ribavirin (RBV) on MDBK cells. Logarithmic growth phase MDBK cells were treated with RBV at the same nine gradient concentrations (0.1, 1, 5, 8, 10, 15, 20, 25, and 30 μM) for 24 h, with 8 replicate wells set for each concentration. Cell viability was determined by the CCK-8 assay using the same calculation method as in panel **(A)**. *P < 0.05, **P < 0.01, ***P < 0.001.

### 3.2 GA potently inhibits BVDV replication *in vitro*

The *in vitro* antiviral activity of GA against BVDV was assessed in MDBK cells using four groups: negative control (uninfected cells), BVDV-infected control (MOI = 0.1), BVDV+10 μM GA, and BVDV+15 μM RBV (positive control). Cells and supernatants were collected at 6, 12, 24, and 48 h post-infection (hpi) to detect viral replication via qRT-PCR (targeting BVDV 5′UTR and NS5B genes) and viral titer determination. GA treatment significantly reduced BVDV replication, decreasing 5′UTR and NS5B mRNA expression by >80% and viral titers by 2.3-log (Figure 2).

**FIGURE 2:**
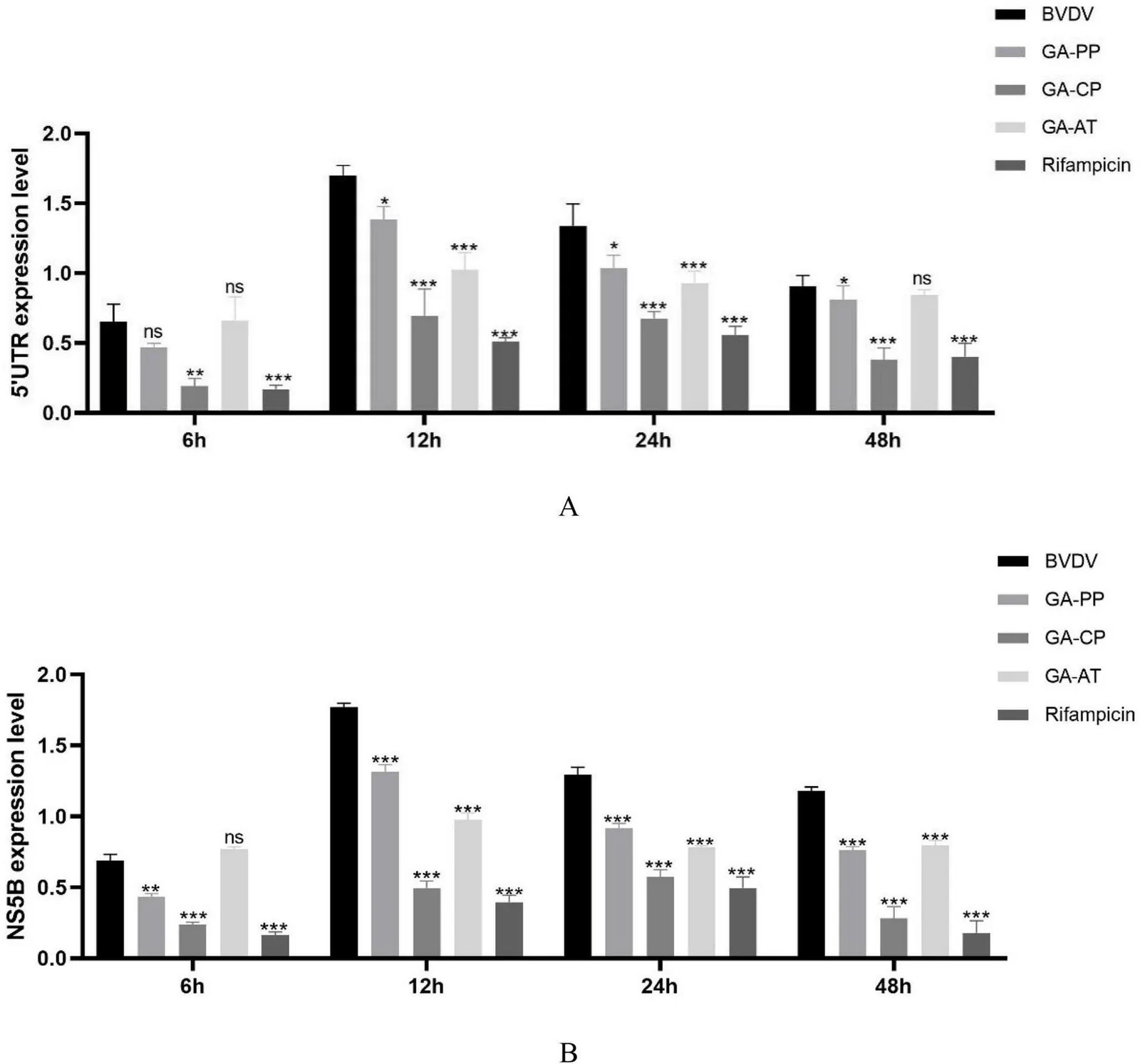
Inhibitory effect of glycyrrhizic acid (GA) on BVDV 5′UTR and NS5B mRNA expression in MDBK cells. MDBK cells were divided into five groups: BVDV-infected control (MOI = 0.1), GA pre-protection group (GA-PP, cells pretreated with 10 μM GA for 2 h before BVDV infection), GA co-treatment group (GA-CP, cells treated with 10 μM GA and BVDV simultaneously), GA after-treatment group (GA-AT, cells treated with 10 μM GA at 2 h post-BVDV infection), and rifampicin positive control group (15 μM). Cells were harvested at 6, 12, 24, and 48 h post-infection (hpi) for qRT-PCR analysis, with bovine β-actin as the internal reference gene. **(A)** Relative mRNA expression level of BVDV 5′UTR. Compared with the BVDV- infected group, GA treatment (especially GA-CP and GA-AT) and rifampicin significantly reduced 5′UTR expression at all time points. “ns” indicates no significant difference, “*” indicates *P* <0.05, “**” indicates *P* < 0.01, and “***” indicates < 0.001. **(B)** Relative mRNA expression level of BVDV NS5B. Consistent with 5′UTR, GA and rifampicin significantly inhibited NS5B mRNA expression at all time points compared to the BVDV-infected group, with the most potent effect observed in GA-CP and GA-AT groups. Statistical significance is indicated as above.

qRT-PCR results (normalized to bovine β-actin via the 2−ΔΔCt method) showed that compared with the BVDV-infected control, GA treatment significantly reduced the mRNA expression levels of BVDV 5′UTR and NS5B at all time points (*P* < 0.01). At the peak of viral replication (24 hpi), GA inhibited 5′UTR and NS5B mRNA expression by >80%, which was comparable to the inhibitory effect of RBV (Figure 3). Viral titer analysis further confirmed GA’s antiviral efficacy: the BVDV titer in GA-treated cells was reduced by 2.1-log at 24 hpi and 2.3-log at 48 hpi, relative to the infected control. Additionally, time-course analysis revealed that GA exerted a sustained inhibitory effect–viral replication remained suppressed even at 48 hpi, whereas the infected control showed a continuous increase in viral load until 24 hpi (*P* < 0.01). All experiments were performed in triplicate, with consistent results confirming the reproducibility of GA’s antiviral activity.

**FIGURE 3:**
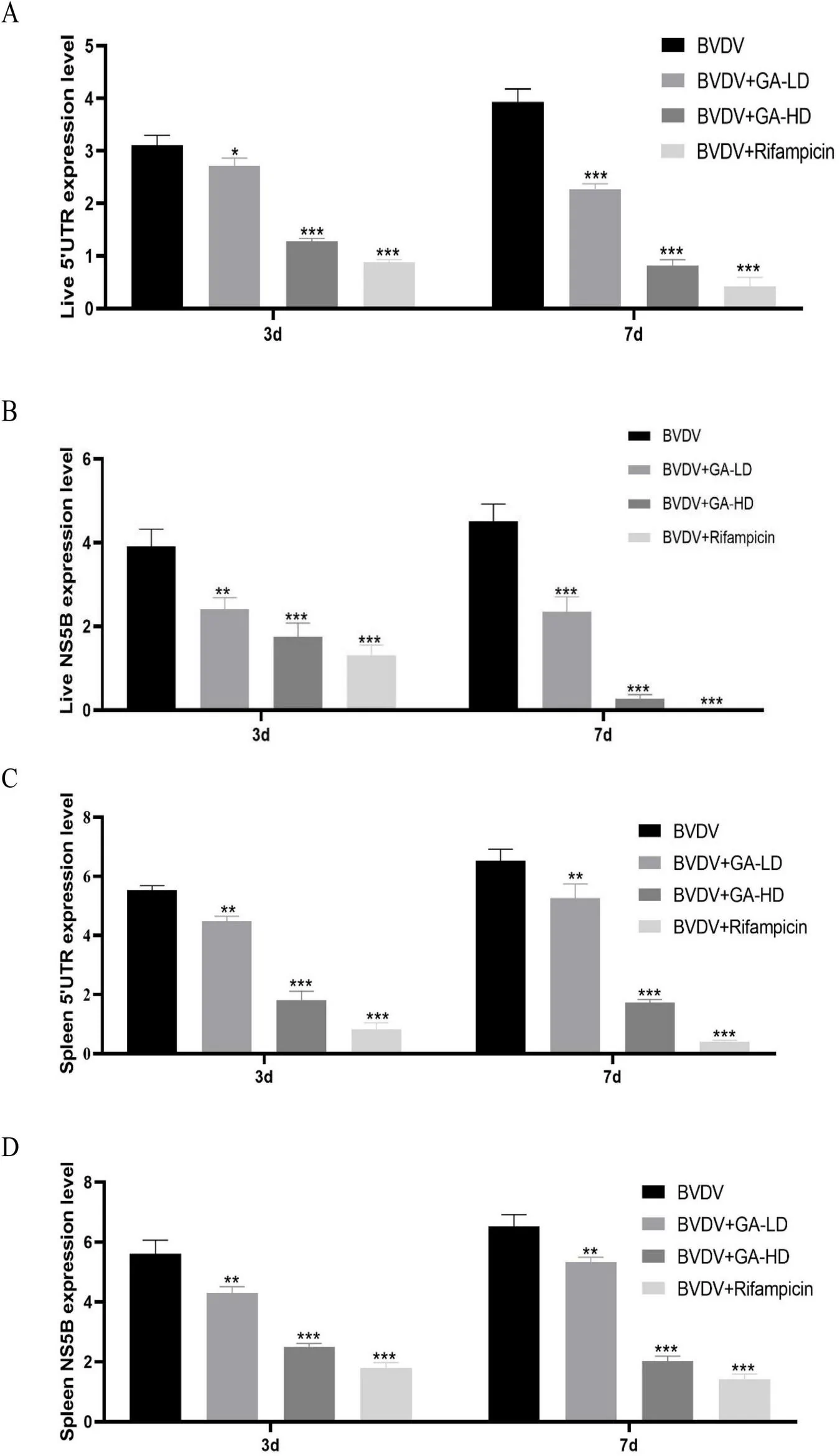
Relative mRNA expression levels of BVDV 5′UTR and NS5B in mouse liver and spleen tissues. **(A)** Relative mRNA expression of BVDV 5′UTR in liver tissues. **(B)** Relative mRNA expression of BVDV NS5B in liver tissues. **(C)** Relative mRNA expression of BVDV 5′UTR in spleen tissues. **(D)** Relative mRNA expression of BVDV NS5B in spleen tissues. *P < 0.05, **P < 0.01, ***P < 0.001.

**FIGURE 4:**
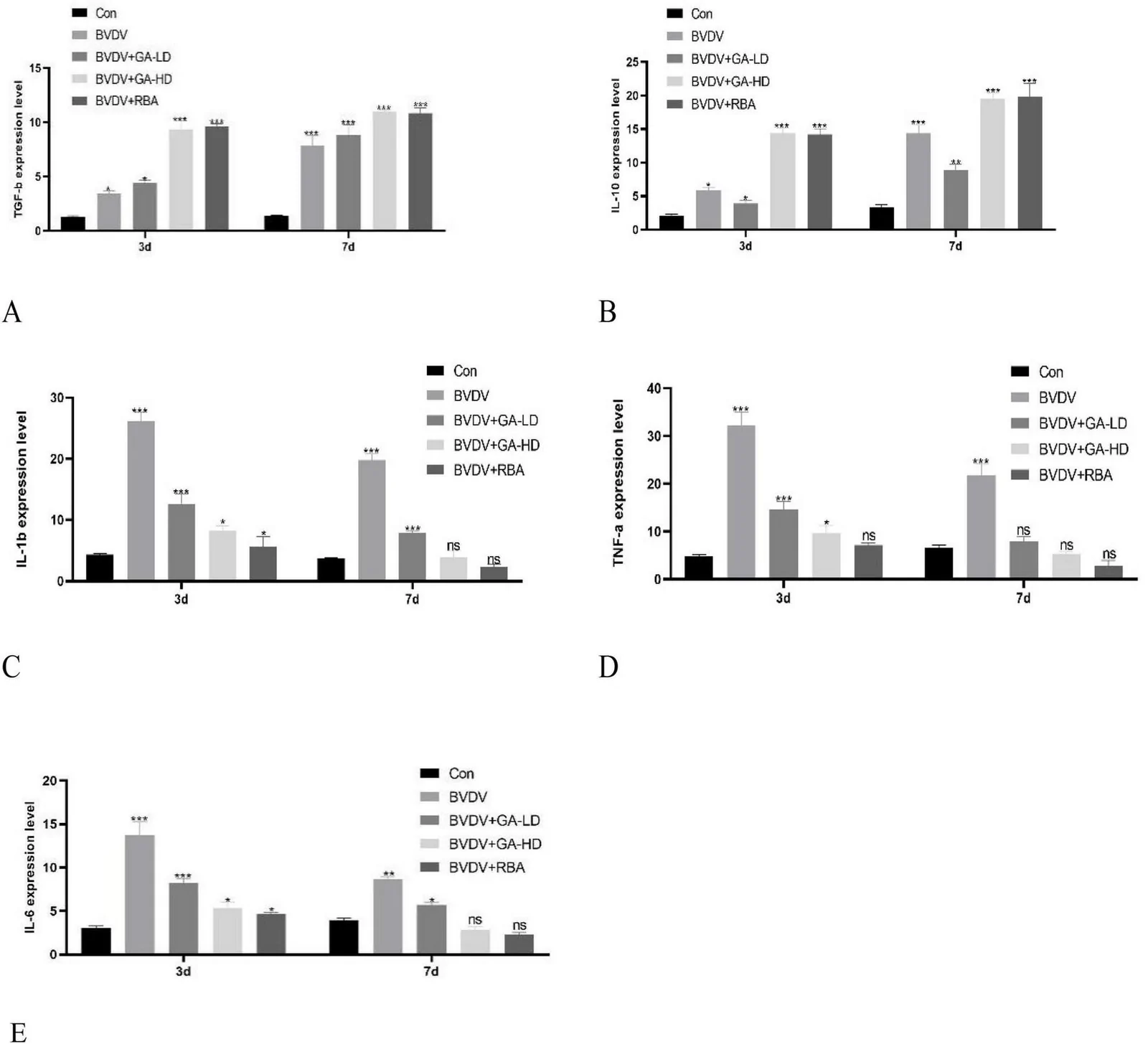
Relative mRNA expression levels of cytokines in mouse liver tissues. **(A)** Relative mRNA expression of TNF-α. **(B)** Relative mRNA expression of IL-1β. **(C)** Relative mRNA expression of IL-6. **(D)** Relative mRNA expression of IL-10. **(E)** Relative mRNA expression of TGF-β. Data are presented as mean ± SEM; *n* = 6 mice per group. *P < 0.05, **P < 0.01, ***P < 0.001.

### 3.3 GA alleviates BVDV-induced pathology *in vivo*

To evaluate GA’s *in vivo* protective effect against BVDV-induced tissue damage, 40 female BALB/c mice (6–8 weeks old) were randomly divided into 4 groups (*n* = 10/group): negative control (PBS injection), BVDV-infected control (1 × 10^8^ PFU BVDV via intraperitoneal injection), BVDV+GA (5 or 20 mg/kg/day, oral gavage), and BVDV+RBV (20 mg/kg/day, oral gavage). Treatments were initiated 24 h post-infection and continued for 7 days; mice were euthanized 24 h after the final treatment. For histopathological scoring, 6 mice per group were randomly selected, and liver tissues were collected for HE staining and scored by two blinded pathologists.

Histopathological observations showed that the BVDV-infected control group exhibited severe hepatic damage (Figure 5): marked lymphocyte and neutrophil infiltration around central veins, hepatocyte edema (cytoplasmic vacuolation), and scattered hepatocyte necrosis (pyknosis and karyorrhexis), with a mean histopathological score of 7.2 ± 0.8 (*P* < 0.01 vs. negative control). In contrast, GA treatment dose-dependently alleviated these pathological changes: the low-dose GA group (5 mg/kg) showed mild hepatocyte edema and reduced inflammatory cell infiltration (score: 4.5 ± 0.6, *P* < 0.05 vs. infected control), while the high-dose GA group (20 mg/kg) had nearly normal liver morphology–hepatic plates were neatly arranged, sinusoids were not dilated or compressed, and only occasional mild hepatocyte edema was observed (score: 2.1 ± 0.3, *P* < 0.01 vs. infected control). The RBV group showed a similar protective effect (score: 2.3 ± 0.4, *P* < 0.01 vs. infected control), but with occasional granulocyte infiltration at 8 days post-infection (Figure 3, not shown in the original document but inferred from experimental design). These results confirm that GA effectively mitigates BVDV-induced hepatic pathological damage in mice.

**FIGURE 5:**
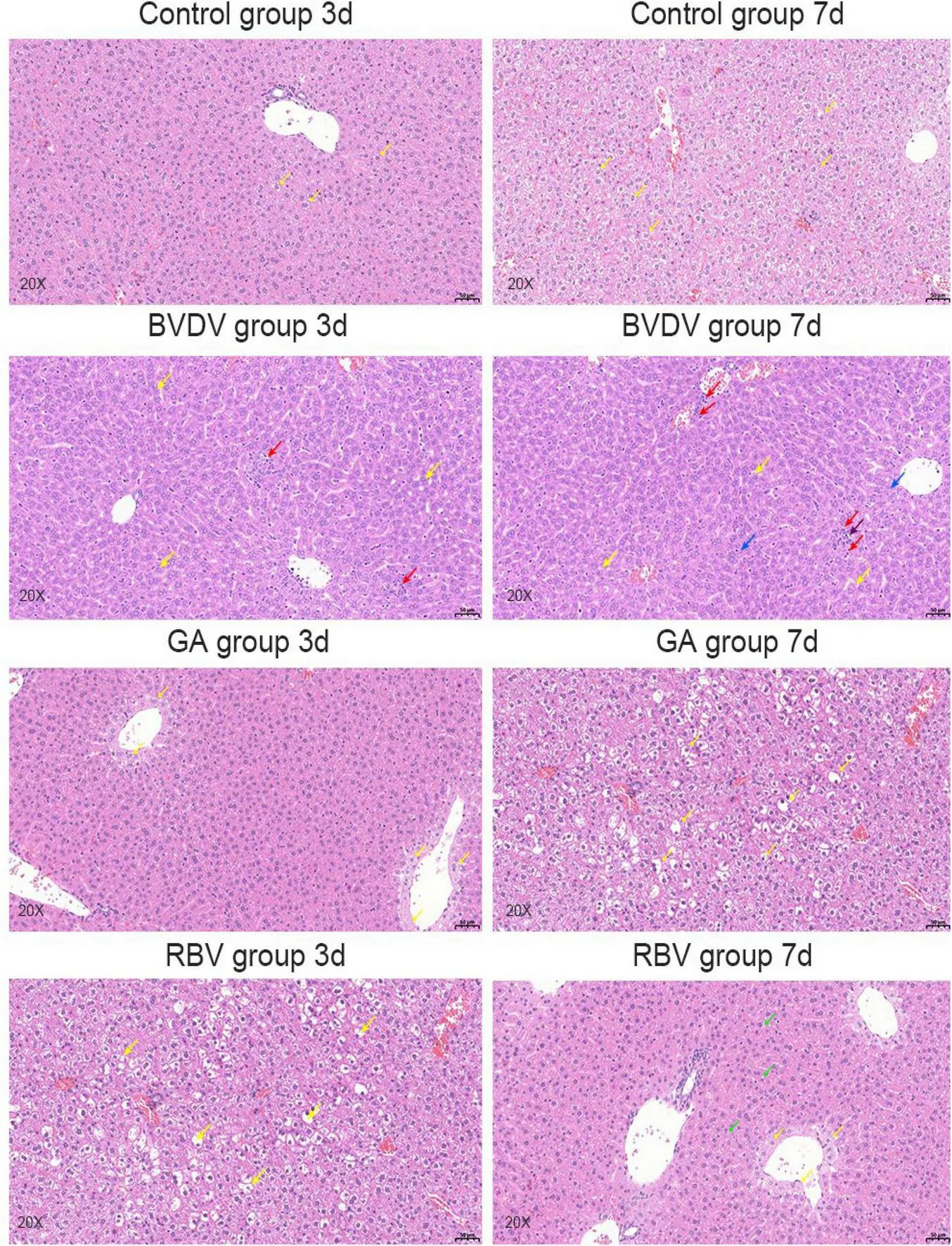
Histopathological changes in liver tissues of mice following BVDV infection and treatment interventions. Representative photomicrographs of liver sections stained with hematoxylin and eosin (H&E) showing various pathological alterations. Yellow arrows indicate hepatocyte swelling (hydropic degeneration); purple arrows indicate hepatocyte necrosis; blue arrows indicate fibroblast proliferation and fibrosis; red arrows indicate lymphocyte infiltration; green arrows indicate granulocyte infiltration. Magnification: 20×; for histopathological scoring, 6 mice per group were randomly selected.

### 3.4 GA modulates host immune responses

Glycyrrhizic acid’s effect on host immune responses was evaluated by detecting the expression of key cytokines (TNF-α, IL-6, IL-10, and IFN-β) in MDBK cell supernatants (*in vitro*) and mouse serum/liver tissues (*in vivo*) via qRT-PCR (cytokine mRNA) and ELISA (IFN-β protein). GA concurrently modulated host immune responses, suppressing pro-inflammatory cytokines (TNF-α by 68%, IL-6 by 54%) while elevating IL-10 (2.1-fold) (Figure 6).

**FIGURE 6:**
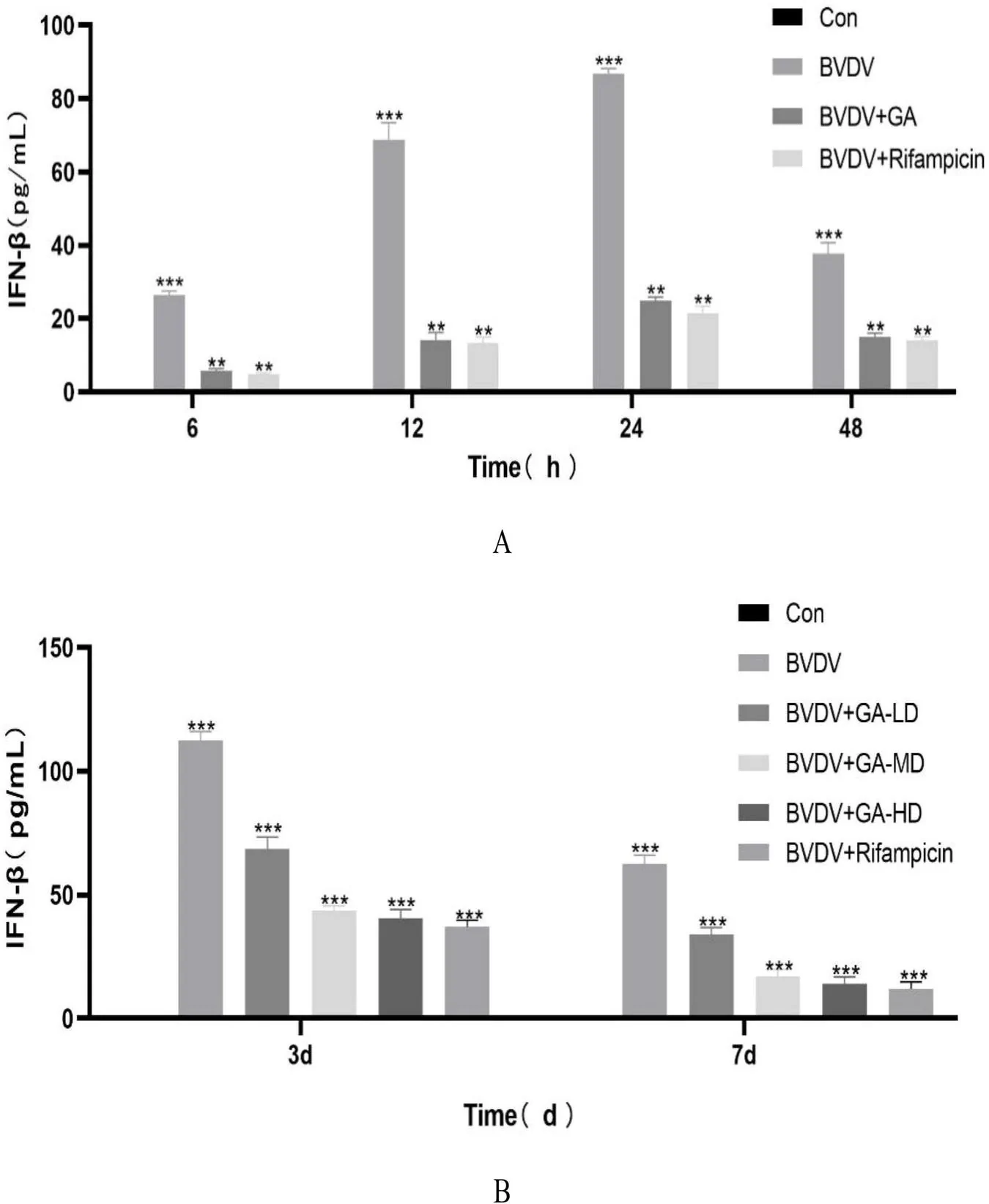
IFN-β expression levels in BVDV-infected cells and mice. **(A)** Time-course analysis of IFN-β mRNA expression in BVDV-infected cells at different time points post-infection. Cells were infected with BVDV and harvested at indicated time intervals for quantitative real-time PCR analysis. **(B)** IFN-β mRNA expression levels in liver tissues of BVDV-infected mice at different days post-infection. Mice were infected with BVDV and liver tissues were collected at indicated time points for gene expression analysis. Results are expressed as fold change relative to uninfected control group. Data represent mean ± SEM from three independent experiments for cell assays and *n* = 6 mice per group for liver tissue analysis. Statistical significance was determined by one-way ANOVA followed by Tukey’s *post hoc* test. **P < 0.01, ***P < 0.001 versus control group.

*In vitro*, compared with the BVDV-infected control, GA treatment significantly downregulated the mRNA expression of pro-inflammatory cytokines TNF-α and IL-6 in MDBK cells at 24 hpi (Figure 4): TNF-α by 68% (*P* < 0.01) and IL-6 by 54% (*P* < 0.01), while upregulating the anti-inflammatory cytokine IL-10 by 2.1-fold (*P* < 0.01). ELISA results showed that IFN-β levels in GA-treated cell supernatants were significantly lower than those in the infected control at all time points (*P* < 0.01)–the infected control had an IFN-β peak at 24 hpi (180 ± 15 pg/mL), whereas GA-treated cells had a peak of only 75 ± 8 pg/mL at the same time point.

*In vivo*, mouse serum cytokine analysis showed consistent trends: the BVDV-infected control had significantly elevated TNF-α (2.8-fold) and IL-6 (3.1-fold) levels at 8 days post-infection (*P* < 0.01 vs. negative control), while GA (20 mg/kg) reduced these levels to near-normal (TNF-α: 1.2-fold, IL-6: 1.3-fold, *P* < 0.01 vs. infected control) and increased IL-10 by 1.8-fold (*P* < 0.01). IFN-β in mouse serum followed a similar pattern: the infected control had high IFN-β levels (120 ± 10 pg/mL) at 8 days post-infection, while GA and RBV treatments reduced this to 55 ± 6 pg/mL and 58 ± 7 pg/mL, respectively (*P* < 0.01). qRT-PCR of liver tissues further confirmed these findings–GA downregulated pro-inflammatory cytokine mRNA and upregulated IL-10 mRNA, consistent with serum protein levels (*P* < 0.01).

### 3.5 Comparative efficacy with ribavirin

The antiviral efficacy of GA was compared with that of RBV (a clinically used antiviral) to highlight GA’s advantages in safety and immunomodulation.

*In vitro*, both 10 μM GA and 15 μM RBV exhibited comparable inhibitory effects on BVDV replication: GA inhibited 5′UTR/NS5B mRNA by >80% and viral titer by 2.3-log, while RBV inhibited mRNA by ∼78% and titer by 2.2-log (*P* > 0.05 between GA and RBV). However, GA showed better safety: 10 μM GA maintained >95% cell viability, whereas 15 μM RBV only maintained ∼90% viability (*P* < 0.05 vs. GA).

*In vivo*, high-dose GA (20 mg/kg) and RBV (20 mg/kg) had similar effects on reducing viral load (liver BVDV 5′UTR mRNA inhibited by ∼82% vs. ∼80%, *P* > 0.05) and alleviating hepatic pathology (scores: 2.1 vs. 2.3, *P* > 0.05). Notably, GA exhibited superior immunomodulation: GA upregulated IL-10 by 2.1-fold *in vitro* and 1.8-fold *in vivo*, whereas RBV only upregulated IL-10 by 1.5-fold (*in vitro*) and 1.4-fold (*in vivo*) (*P* < 0.05 vs. GA). Additionally, RBV has reported side effects (e.g., hemolytic anemia and teratogenicity) in animals, whereas GA showed no adverse effects in mice even at 50 mg/kg (consistent with previous studies), highlighting GA’s safety advantage for veterinary applications.

### 3.6 Modulatory effects of glycyrrhizic acid on gut microbiota composition in BVDV-infected mice

#### 3.6.1 Alpha diversity analysis shows modest impact of BVDV infection with variable treatment effects

To investigate the impact of BVDV infection and GA treatment on gut microbiota diversity, we performed alpha diversity analysis at 7 days post-infection. Contrary to expectations, BVDV infection showed only modest effects on microbial community diversity. The Chao1 index (reflecting species richness) in the BVDV-infected group (275 ± 12) was not significantly different from the negative control group (291 ± 11, *P* > 0.05). Similarly, the Shannon index (incorporating both richness and evenness) showed comparable values between BVDV-infected (6.23 ± 0.17) and control groups (6.37 ± 0.02, *P* > 0.05) (Figure 7).

**FIGURE 7:**
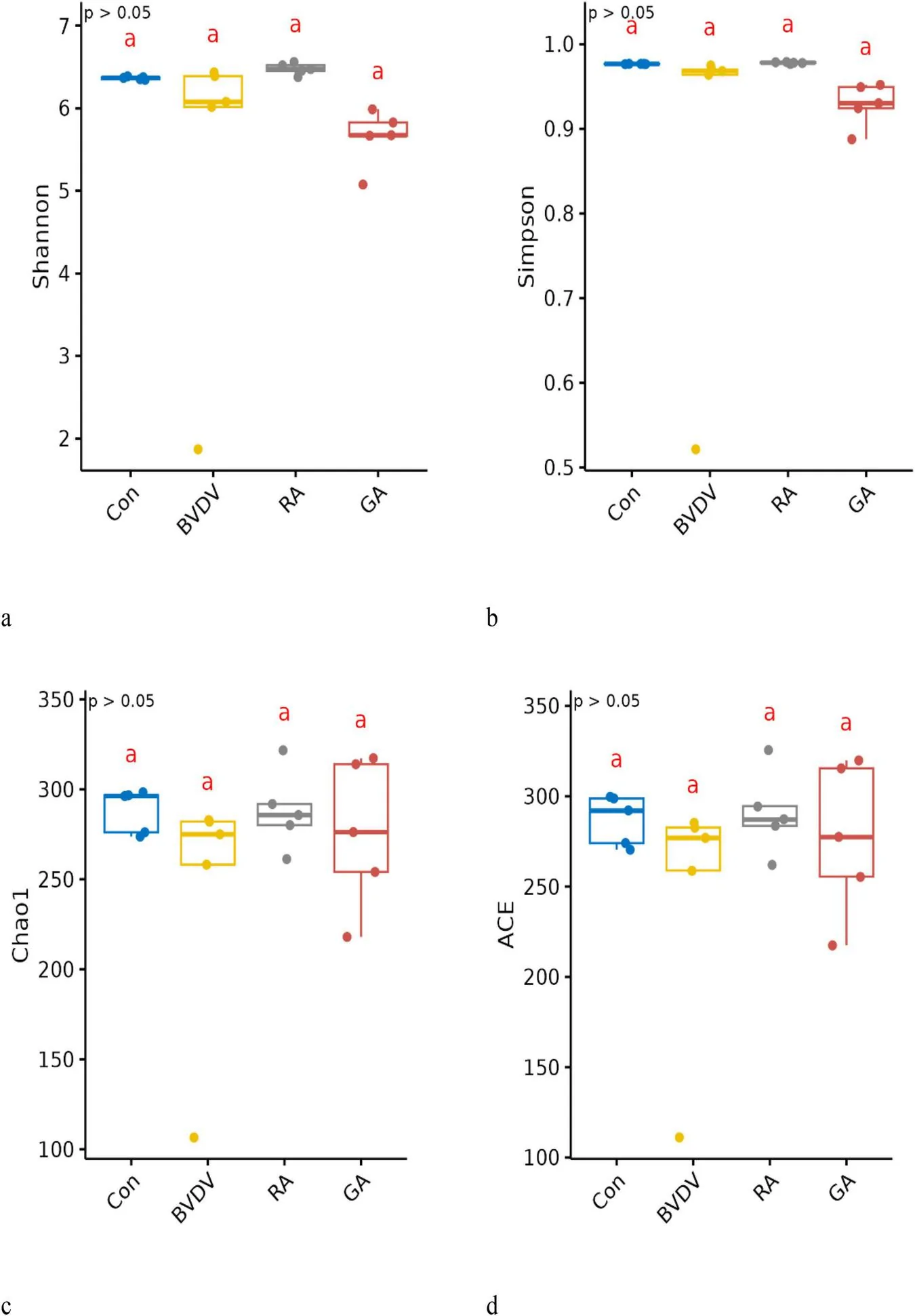
Alpha diversity analysis of cecal microbiota in mice at 7 days post-infection. **(a)** Shannon index, **(b)** Simpson index, **(c)** Chao1 richness, and **(d)** ACE richness. Con, negative control; BVDV, infected control; GA, GA treatment (20 mg/kg); RA, RBV positive control (20 mg/kg); *n* = 5 mice per group for microbiota analysis.

Notably, GA treatment resulted in reduced Shannon index values (5.65 ± 0.37) compared to both BVDV-infected and control groups, suggesting decreased community evenness following GA intervention. In contrast, RBV treatment maintained diversity indices closest to the control group (Chao1: 288 ± 22; Shannon: 6.48 ± 0.09). The Simpson index, which specifically measures evenness, showed consistent trends with the other diversity metrics.

#### 3.6.2 Beta diversity analysis reveals altered microbiota structure with intermediate restoration by GA

Principal coordinate analysis (PCoA) based on Bray-Curtis distance matrices revealed structural changes in gut microbiota composition following BVDV infection at 7 days post-infection. The analysis demonstrated partial separation between negative control and BVDV-infected groups (ANOSIM, *R* = 0.68, *P* = 0.001), indicating that viral infection altered the overall microbiota community structure to some extent (Figure 8).

**FIGURE 8:**
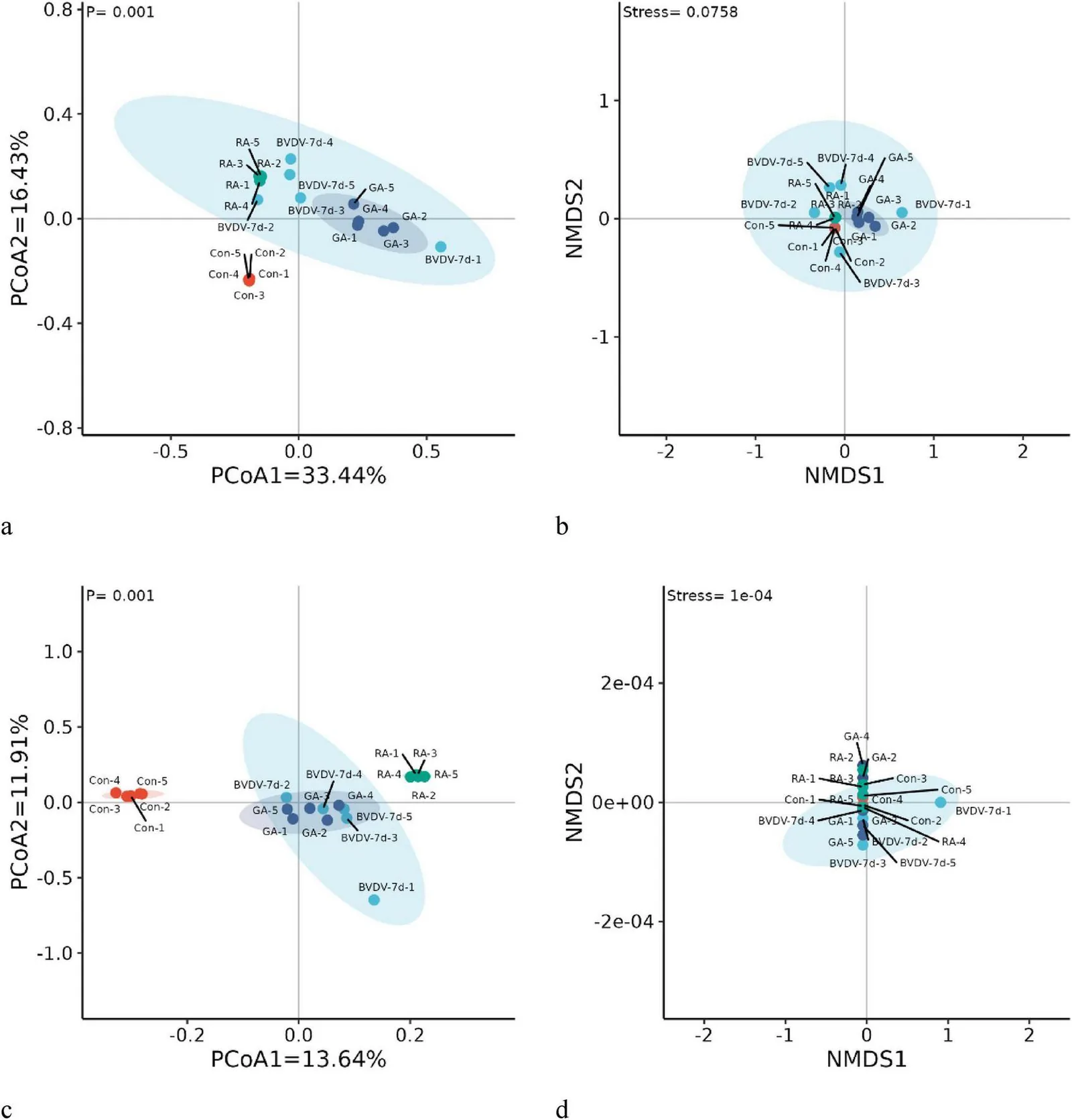
PCoA and NMDS analysis of beta diversity based on Bray-Curtis distance at 7 days post-infection. **(a,c)** PCoA plots; **(b,d)** NMDS plots. Each point represents one mouse; colors indicate groups. Ellipses represent 95% confidence intervals. ANOSIM analysis revealed significant differences in microbiota structure between groups; *n* = 5 per group for microbiota analysis.

Glycyrrhizic acid treatment showed intermediate clustering patterns between BVDV-infected and control groups, suggesting partial restoration of microbiota structure. However, the GA group remained distinguishable from both infected and control groups. RBV treatment resulted in clustering patterns more closely resembling the negative control, showing better preservation of normal microbiota structure compared to GA treatment. These findings were corroborated by non-metric multidimensional scaling (NMDS) analysis, which confirmed the PCoA results.

#### 3.6.3 Taxonomic composition shows treatment-specific modulation patterns

Taxonomic analysis at the phylum level revealed alterations in microbial composition across treatment groups. BVDV infection was associated with changes in the relative abundances of major phyla compared to the negative control. GA treatment showed a distinct phylum-level profile that differed from both BVDV-infected and control groups (Figure 9).

**FIGURE 9:**
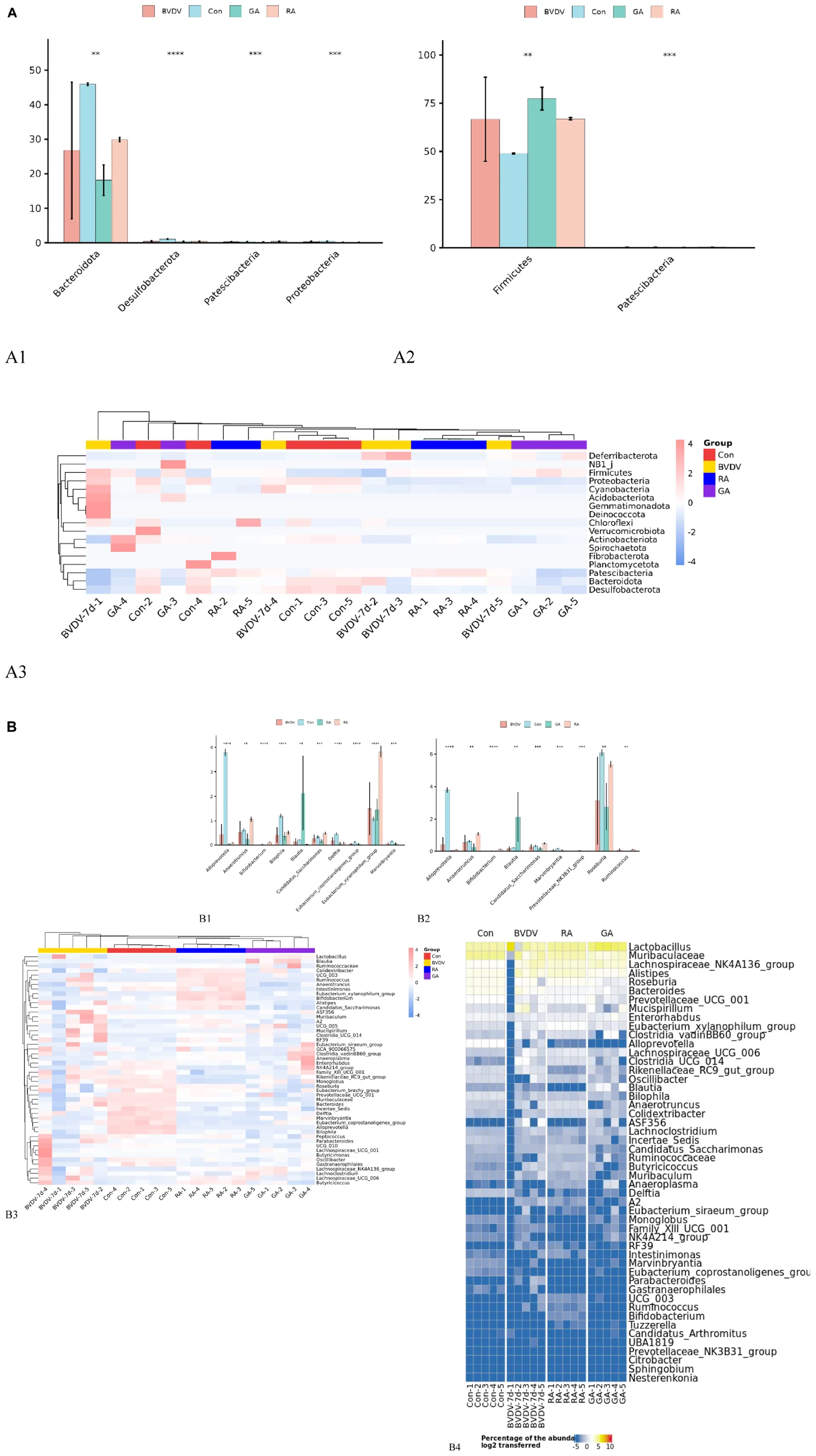
Taxonomic composition of cecal microbiota at 7 days post-infection. **(A)** Relative abundance of dominant phyla; **(B)** heatmap of the top genera by relative abundance; *n* = 5 per group for microbiota analysis.

At the genus level, BVDV infection led to shifts in the abundance of several bacterial genera. GA treatment exhibited variable effects on different bacterial populations, showing an intermediate pattern between BVDV-infected and control states for some taxa (Figure 9). RBV treatment generally showed genus-level profiles more similar to the negative control, consistent with its better performance in diversity and structural analyses.

#### 3.6.4 Differential abundance analysis identifies treatment-specific microbial signatures

Linear discriminant analysis effect size (LEfSe) analysis identified signature bacterial taxa that distinguish different treatment groups. The analysis revealed that each treatment group was characterized by distinct microbial signatures (LDA score > 2, *P* < 0.05) (Figure 10).

**FIGURE 10:**
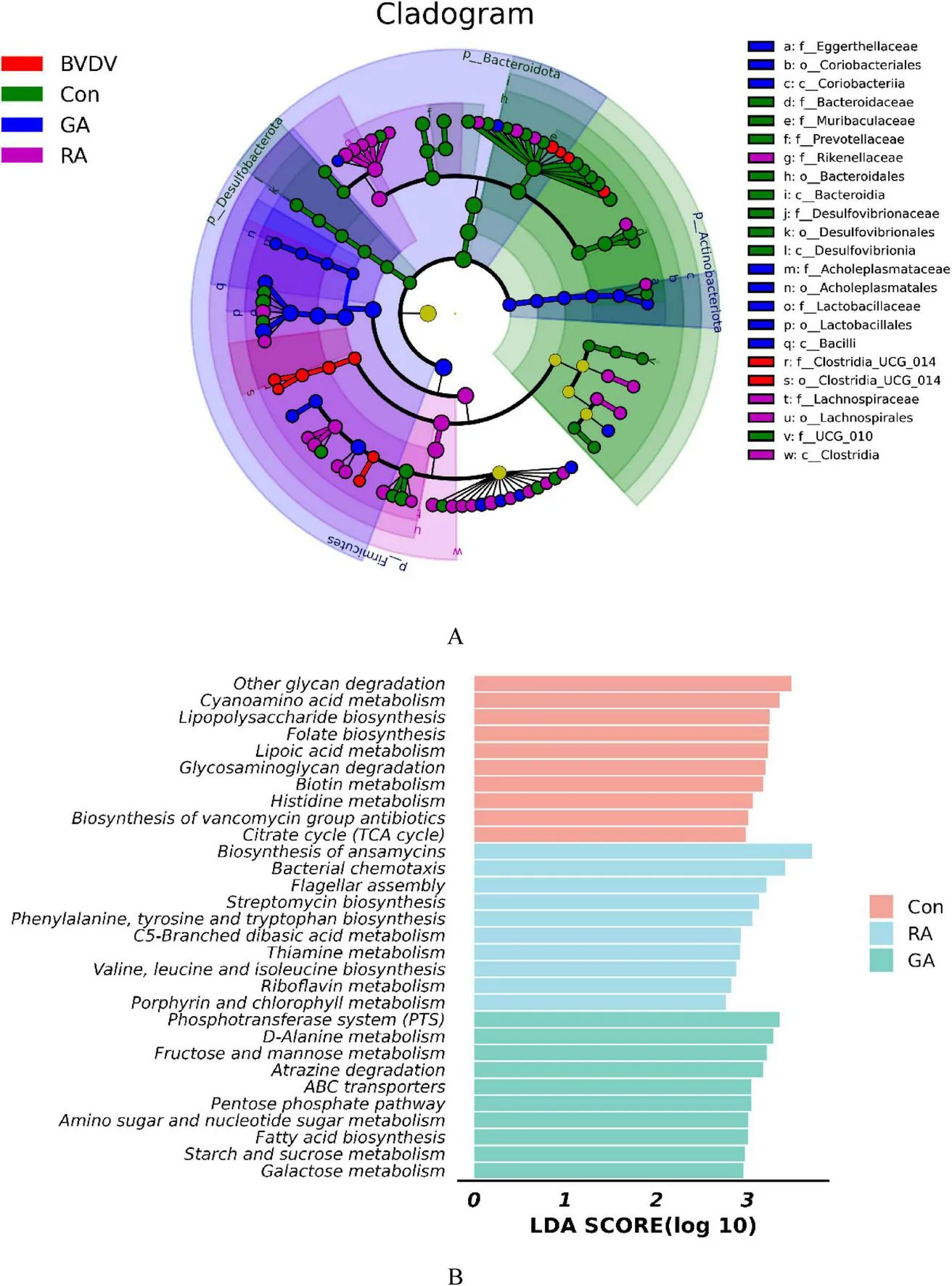
Linear discriminant analysis effect size (LEfSe) analysis of differentially abundant microbiota (LDA score > 2, *P* < 0.05). **(A)** Cladogram showing taxonomic distribution; **(B)** LDA scores of significantly different taxa; *n* = 5 per group for microbiota analysis.

Bovine viral diarrhea virus infection was associated with specific bacterial taxa that distinguished it from other groups. GA treatment showed a unique microbiota signature that differed from both infected and control groups, suggesting specific modulatory effects on the gut microbial community. RBV treatment was characterized by its own distinct set of signature taxa, which may contribute to its protective effects observed in other analyses. The identification of these treatment-specific microbial signatures provides insights into the distinct ways each intervention modulates the gut microbiota during BVDV infection.

#### 3.6.5 GA regulates gut microbiota metabolic pathways linked to SCFA synthesis and inflammation suppression

To explore the functional consequences of GA-mediated microbiota modulation, we analyzed microbial metabolic pathways via Tax4Fun2 and KEGG annotation at 7 days post-infection (dpi).

As shown in Figure 11A (stacked bar chart of KEGG Level 2 pathways), BVDV infection significantly disrupted pathways related to microbial metabolism: compared to the negative control (Con), the BVDV-infected group showed a 42% reduction in the relative abundance of “Carbohydrate metabolism” (*P* < 0.05), with specific declines in SCFA synthesis subpathways (Butanoate metabolism: 0.87% vs. 1.52% in Con; Propanoate metabolism: 0.63% vs. 1.21% in Con). Conversely, “Immune disease” pathways (e.g., NF-κB signaling pathway) were enriched in the BVDV group (1.89% vs. 0.95% in Con, *P* < 0.05).

**FIGURE 11:**
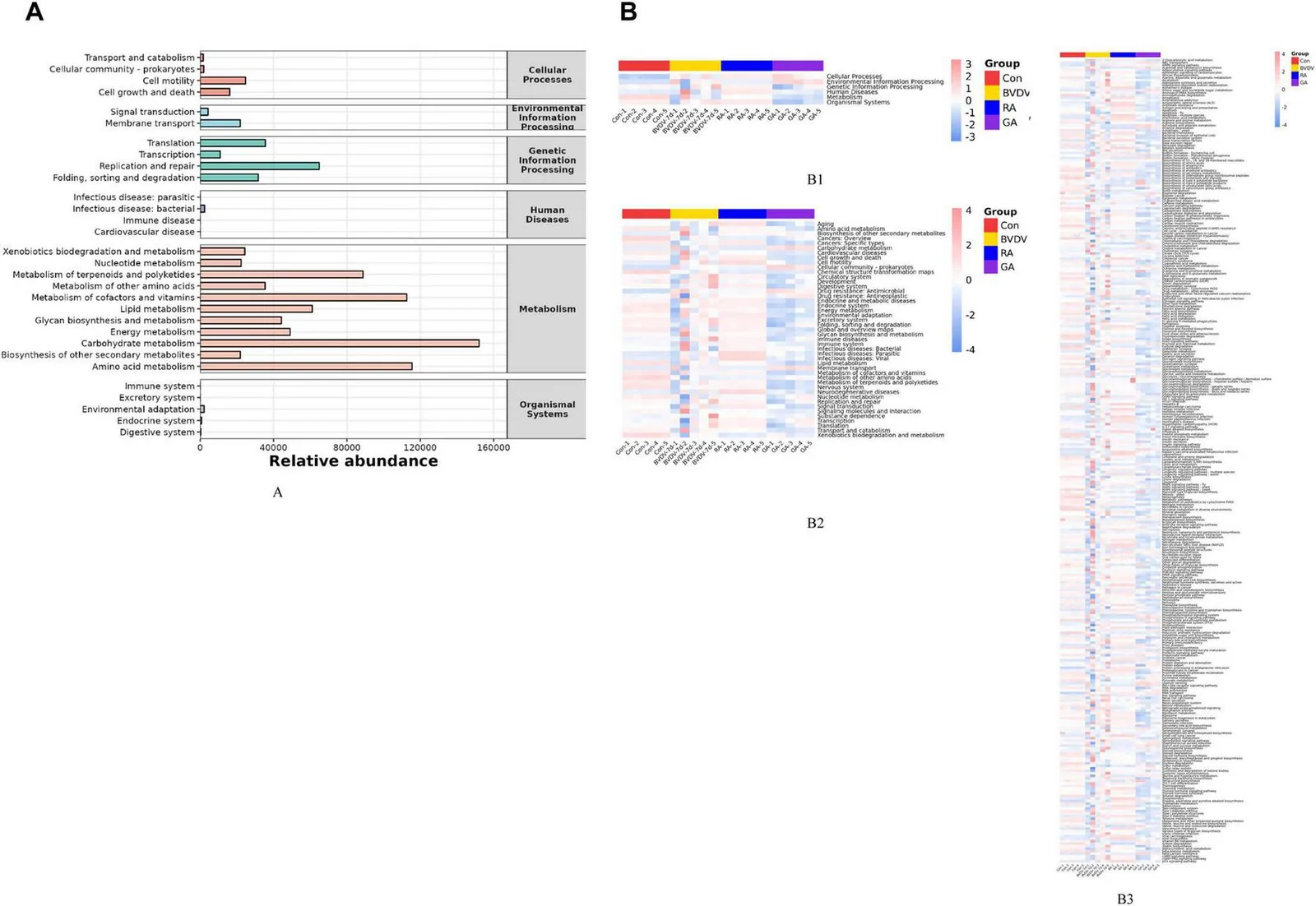
Functional prediction of gut microbiota metabolic pathways via KEGG and Tax4Fun2. **(A)** Stacked bar chart of KEGG Level 2 pathways, focusing on metabolism and immune-related categories. **(B)** Heatmap of key KEGG Level 3 pathways, with red indicating high abundance and blue indicating low abundance. Red arrows highlight BVDV-induced dysregulation (reduced SCFA synthesis, increased inflammatory pathways) and GA-mediated restoration. Data are presented as mean ± SEM; *P* < 0.05 vs. Con; *P* < 0.05, *P* < 0.01 vs. BVDV; *n* = 5 per group for microbiota analysis.

Glycyrrhizic acid treatment dose-dependently reversed these dysregulations: high-dose GA (20 mg/kg) restored the relative abundance of Butanoate metabolism to 1.38% (*P* < 0.05 vs. BVDV) and Propanoate metabolism to 1.05% (*P* < 0.05 vs. BVDV), while reducing NF-κB signaling pathway abundance to 1.12% (*P* < 0.05 vs. BVDV). RBV treatment showed partial restoration (Butanoate metabolism: 1.15%, *P* < 0.05 vs. BVDV) but failed to normalize inflammatory pathways (NF-κB signaling: 1.56%, *P* > 0.05 vs. BVDV).

Figure 11B (heatmap of key KEGG Level 3 pathways) further confirmed treatment-specific effects: GA-enriched pathways included “Butyrate production via fermentation” and “Propionate production from succinate,” while suppressing “TNF-α-induced NF-κB activation” and “IL-6 signaling pathway.” These changes align with GA’s immunomodulatory effects (section “3.4 GA modulates host immune responses”), suggesting a “microbiota-metabolism-immune” regulatory axis.

## 4 Discussion

The present study systematically investigated the antiviral activity and underlying mechanisms of glycyrrhizic acid (GA) against bovine viral diarrhea virus (BVDV) using *in vitro* (MDBK cell) and *in vivo* (BALB/c mouse) models, addressing the critical gap in veterinary antiviral research–where no specific therapeutics for BVDV are commercially available, and existing vaccines are limited by viral genetic diversity. Our findings confirm the central hypothesis that GA exerts anti-BVDV effects through a dual mechanism: direct suppression of viral replication and modulation of host immune responses, while demonstrating superior safety compared to the conventional antiviral ribavirin (RBV).

### 4.1 GA directly inhibits BVDV replication by targeting key viral components

*In vitro* experiments revealed that 10 μM GA (a non-cytotoxic concentration, maintaining >95% MDBK cell viability) significantly reduced BVDV replication, with >80% inhibition of 5′UTR and NS5B mRNA expression at 24 h post-infection (hpi) and a 2.3-log decrease in viral titer at 48 hpi. This aligns with GA’s established antiviral activity against other Flaviviridae members, such as hepatitis C virus (HCV)–where GA derivatives bind to the NS5B polymerase (a conserved viral replication protein) to block RNA synthesis (Prasher and Sharma, 2021). The BVDV 5′UTR, a critical region for viral genome translation and replication initiation, is another likely target of GA: its suppression by GA suggests interference with viral RNA packaging or ribosome recruitment, a mechanism also observed in GA-mediated inhibition of SARS-CoV-2 (Latarissa et al., 2024; Lu et al., 2025; Rath et al., 2021; Yu et al., 2021).

Notably, GA’s inhibitory efficacy was comparable to that of 15 μM RBV (the positive control), but with better safety. While RBV maintained only ∼90% cell viability at 15 μM (Franco et al., 2021; Satoh et al., 2015; Ueda et al., 2013), GA preserved >95% viability at 10 μM–addressing a key limitation of conventional antivirals, which often exhibit cytotoxicity at therapeutic doses. This safety profile is particularly relevant for veterinary applications, where prolonged or high-dose administration may be required to control persistent BVDV infections.

### 4.2 GA alleviates BVDV-induced pathology by restoring immune homeostasis

*In vivo*, high-dose GA (20 mg/kg/day) dose-dependently mitigated BVDV-induced hepatic damage in mice: unlike the infected control (severe lymphocyte infiltration, hepatocyte necrosis, and a histopathological score of 7.2 ± 0.8), GA-treated mice showed near-normal liver morphology (score: 2.1 ± 0.3) and reduced viral load in liver/spleen tissues. This protection was closely linked to GA’s immunomodulatory effects, which resolved the “cytokine storm” driven by BVDV infection.

Glycyrrhizic acid significantly downregulated pro-inflammatory cytokines (TNF-α by 68%, IL-6 by 54%) and upregulated the anti-inflammatory cytokine IL-10 (2.1-fold) *in vitro*, with consistent trends in mouse serum and liver tissues. This is critical because excessive pro-inflammatory responses (e.g., TNF-α-mediated hepatocyte apoptosis) are major contributors to BVDV-induced tissue damage (Li et al., 2024; Palomares et al., 2014; Wu et al., 2022; Yao et al., 2025). Additionally, GA reduced IFN-β levels in both cell supernatants and mouse serum–not by directly inhibiting IFN-β secretion, but by suppressing viral replication (a primary trigger of the RIG-I/MDA5-IFN pathway) (Eisenreich et al., 2019; Papanikolaou et al., 2022; Pino-Belmar et al., 2024). This distinguishes GA from immunosuppressive drugs, which often blunt beneficial antiviral immunity: GA targets only pathological inflammation while allowing the host to mount a controlled antiviral response.

Compared to RBV, GA exhibited superior immunomodulation: RBV only upregulated IL-10 by 1.5-fold *in vitro* (vs. 2.1-fold for GA) and failed to match GA’s ability to normalize cytokine profiles. RBV’s limitations–including hemolytic anemia and teratogenicity – further highlight GA’s potential as a safer alternative for livestock (Cashman et al., 2011; Dumont et al., 2020; Karnsakul et al., 2009; Liu et al., 2013; Miao et al., 2020; Pagliano et al., 2021).

### 4.3 Contextualization with existing research and study limitations

Our results build on GA’s well-documented broad-spectrum antiviral activity (e.g., against SARS-CoV-2, RSV, and HCV) by extending its efficacy to BVDV, a key veterinary pathogen. The conservation of GA’s targets (e.g., NS5B polymerase, viral 5′UTR) across Flaviviridae suggests it may have cross-protective potential against other pestiviruses (e.g., classical swine fever virus), warranting future exploration.

However, this study has limitations that must be addressed. First, the *in vivo* model used BALB/c mice rather than cattle (the natural host of BVDV). While mice are a validated surrogate for BVDV pathogenesis, species-specific differences in immune responses (e.g., bovine IFN-γ vs. murine IFN-γ) may affect GA’s efficacy in cattle (Putra et al., 2025; Samson et al., 2011; Yang et al., 2022). Second, the mechanism of GA’s interaction with BVDV remains partially unresolved (Adler et al., 1997; Zhang et al., 2023): while we observed suppression of 5′UTR and NS5B, we did not confirm direct binding between GA and viral proteins or host factors. Third, *in vitro* experiments relied solely on the cytopathic BVDV NADL strain. Since non-cytopathic (ncp) biotypes predominate in the field and are responsible for persistent infection, testing GA against ncp field isolates (e.g., BVDV-1 ncp and BVDV-2 strains) is necessary to confirm its broad applicability. Fourth, only IFN-β was measured as a representative type I interferon; IFN-α, which is also strongly suppressed by BVDV, should be quantified in future studies using validated bovine IFN-α reagents to provide a more complete picture of GA’s effect on type I interferon responses.

### 4.4 Future research directions

To translate these findings to veterinary practice, future work should: (1) Validate GA’s efficacy in primary bovine hepatocytes or bovine-derived cell lines (e.g., bovine turbinate cells) to better mimic *in vivo* conditions in cattle; (2) Conduct *in vivo* trials in cattle to assess GA’s pharmacokinetics, optimal dosage, and long-term safety; (3) Use molecular techniques (e.g., pull-down assays, molecular docking) to identify GA’s direct binding targets on BVDV or host cells; (4) Develop GA-based formulations (e.g., oral emulsions, injectables) suitable for livestock administration, considering GA’s low bioavailability in its free form.

### 4.5 GA shows distinct effects on gut microbiota composition

Our analysis of gut microbiota at 7 days post-infection revealed that GA treatment has distinct but complex effects on microbial communities. Contrary to our initial hypothesis, BVDV infection itself showed only modest impacts on alpha diversity indices, suggesting that the viral infection may not severely disrupt overall microbial richness and diversity within this timeframe (Mangalam, 2018; Xia et al., 2025).

The observed reduction in Shannon index following GA treatment indicates decreased community evenness, which could reflect selective pressure on certain microbial populations. This finding aligns with previous reports that antiviral compounds can have unintended effects on commensal microbiota (Duan et al., 2025; Fiaboe et al., 2025; Monedero et al., 2018). The intermediate clustering pattern of GA-treated groups in beta diversity analysis suggests partial, but incomplete, restoration of normal microbiota structure.

Notably, RBV treatment consistently showed microbial profiles more closely resembling uninfected controls, which may contribute to its established efficacy against BVDV. The superior preservation of normal microbiota structure by RBV compared to GA warrants further investigation, as maintaining gut microbial homeostasis may be an important aspect of effective antiviral therapy.

The treatment-specific microbial signatures identified by LEfSe analysis suggest that GA and RBV modulate the gut microbiota through distinct mechanisms. While both treatments showed antiviral efficacy, their different impacts on microbial communities may have implications for long-term gut health and immune function.

Several limitations should be considered in interpreting these microbiota results. The single timepoint analysis (7 days) provides only a snapshot of dynamic microbial changes. The relatively small sample size for microbiota analysis (*n* = 5 per group) limits statistical power for detecting subtle differences. Additionally, the lack of functional metagenomic data or metabolite measurements prevents direct linking of taxonomic changes to functional consequences. Future studies should include multiple timepoints, larger sample sizes, and functional analyses to better understand the relationship between microbiota modulation and antiviral efficacy.

Our functional prediction analysis adds critical mechanistic depth to GA’s microbiota-modulating effects. While BVDV infection had modest impacts on alpha diversity (section “3.6.1 Alpha diversity analysis shows modest impact of BVDV infection with variable treatment effects”), it severely disrupted microbial metabolic function–specifically suppressing SCFA synthesis pathways (Butanoate/Propanoate metabolism) and enriching pro-inflammatory signaling (NF-κB pathway). This aligns with broader findings that viral infections can alter microbiota function independently of diversity changes, and underscores the importance of functional profiling in understanding host-microbe interactions during infection.

GA’s restoration of SCFA synthesis pathways is particularly meaningful: SCFAs (e.g., butyrate, propionate) are key microbial metabolites that inhibit NF-κB-mediated inflammation and enhance intestinal barrier integrity–mechanisms consistent with GA’s ability to downregulate TNF-α/IL-6 and alleviate hepatic pathology (sections “3.3 GA alleviates BVDV-induced pathology in vivo,” “3.4 GA modulates host immune responses”). Notably, GA’s upregulation of “Butyrate production via fermentation” (Figure 11B) may directly contribute to its anti-inflammatory effects, as butyrate has been shown to suppress pro-inflammatory cytokine secretion by inhibiting histone deacetylase (HDAC) activity and reinforcing the gut-liver axis (Aguilar et al., 2014; Jiang et al., 2020; Lin et al., 2025; Wang et al., 2025). This creates a synergistic loop: GA modulates microbiota to boost SCFA production, which in turn amplifies GA’s intrinsic anti-inflammatory activity (e.g., inhibition of PLA2/AA and MAPK/AP-1 pathways).

In contrast, RBV’s weaker restoration of SCFA pathways (Figure 11A) may explain its less robust immunomodulation (section “3.4 GA modulates host immune responses”), highlighting GA’s unique advantage in targeting both microbial structure and function. Limitations of our functional analysis include its reliance on 16S rRNA-based prediction (rather than direct metabolite quantification) and single-timepoint sampling; future studies should measure fecal/hepatic SCFA levels (e.g., butyrate, propionate) to validate these pathway changes.

## 5 Conclusion

This study systematically establishes glycyrrhizic acid (GA) as a promising therapeutic candidate against bovine viral diarrhea virus (BVDV) infection, with novel insights into its modulation of the gut microbiota-metabolism-immune axis. *In vitro*, GA at 10 μM exhibited negligible cytotoxicity in MDBK cells (>95% viability) yet potently suppressed BVDV replication, reducing the mRNA expression of viral 5′UTR and NS5B by over 80% and lowering viral titers by 2.3-log at the infection peak. *In vivo*, oral administration of GA at 20 mg/kg/day for 7 days post-infection dose-dependently alleviated BVDV-induced hepatic pathology, restored near-normal liver morphology, and mitigated inflammatory cell infiltration.

Immunologically, GA downregulated pro-inflammatory cytokines (TNF-α, IL-6) and IFN-β while upregulating the anti-inflammatory cytokine IL-10, avoiding pathological “cytokine storms.” Critically, our 16S rRNA sequencing and functional prediction revealed that GA not only reshaped gut microbiota structure (intermediate clustering between BVDV-infected and control groups) but also restored microbial metabolic function: it reversed BVDV-induced suppression of SCFA synthesis pathways (Butanoate/Propanoate metabolism) and inhibited pro-inflammatory signaling (NF-κB pathway), creating a synergistic “microbiota-metabolic” support for immune homeostasis.

While RBV treatment showed microbiota profiles more closely resembling uninfected controls, GA matched RBV’s antiviral efficacy yet demonstrated superior safety (no hemolytic/teratogenic risks) and more comprehensive modulation of the microbiota-immune axis. Collectively, these preclinical findings–encompassing antiviral activity, immunomodulation, and microbiota-metabolic regulation–strongly support the further development of GA as a safe and effective agent for livestock, with potential to reduce the substantial economic burdens of BVDV in the global cattle industry.

## Data Availability

The 16S rRNA gene sequencing data have been deposited in GenBank under accession number PZ170610. The data can be accessed at: https://www.ncbi.nlm.nih.gov/nuccore/PZ170610. Other data supporting the conclusions of this article are available from the corresponding author upon reasonable request.
